# Adolescent Klinefelter syndrome: is there an advantage to testis tissue harvesting or not?

**DOI:** 10.12688/f1000research.8395.1

**Published:** 2016-07-06

**Authors:** Robert Oates

**Affiliations:** 1 Boston University School of Medicine, Boston, MA, USA

**Keywords:** testicular sperm extraction, fertility, non-obstructive azoospermic, spermatogenesis

## Abstract

It is currently unclear whether an adolescent with 47,XXY Klinefelter syndrome will be better off having testicular sperm extraction (TESE) performed in an effort to ‘preserve fertility’ for the future or, alternatively, should be advised to simply wait until adulthood when he and his partner are ready to begin a family. This report will provide data suggesting that there is no obvious ‘preservation’ benefit and that recommending TESE to the 47,XXY boy and his parents may not be as helpful as it might appear and may be overly aggressive.

## Introduction

Klinefelter syndrome (KS) (47,XXY) is the most common chromosomal disorder in men, affecting one in 600 newborn males, and is etiologic in up to 11% of non-obstructive azoospermic (NOA) males ^1^. When testicular sperm extraction (TESE) became a standard technique for the discovery and retrieval of sperm in the NOA patient, it was subsequently successfully applied to KS adults, who had previously been considered sterile. Published reports from many different centers describe rates of sperm retrieval in these 47,XXY adults to be approximately 50–70%, along with excellent pregnancy rates and healthy 46,XY or 46,XX offspring, as recently reviewed by Majzoub *et al.* ^2^.

For a variety of reasons, KS may occasionally be diagnosed prenatally, in infants, in young boys, or in adolescent males ^3– 7^. Based upon the knowledge that not all adult KS males will have spermatozoa harvested upon TESE, several clinical investigators have pursued a course of action to determine if TESE should be offered and applied to the 47,XXY adolescent in order to “preserve fertility potential” ^8, 9^. This submission will address this vexing question from many different angles and try to answer whether this is an overly aggressive approach with more harm than benefit or if this is an appropriate strategy with more benefit than harm.

The underlying tenets for recommending TESE in adolescent KS males is that fertility potential and the presence of testicular spermatozoa are maximal at puberty or just after, and that both are precipitously and irrevocably lost over the next several years. Is that actually true or has fiction become belief that has become fact that has become dogma? Additionally, some feel that testosterone levels plummet after puberty and require replacement, and TESE should be carried out prior to that. However, in 1985, Saltenblatt concluded that testosterone levels settle at low-normal values for most with KS after puberty and remain at those levels into adulthood ^10^. More recently, Aksglaede *et al.* reviewed 166 non-mosaic KS boys and demonstrated that there is no rapid, inexorable decline in their serum testosterone values (which were also low-normal in most subjects but high-normal in some) spanning early adolescence to adulthood ^11^.

## Why is there spermatogonial cell apoptosis at puberty? Is it helpful to preserve these cells?

At conception, the nascent embryo is 47,XXY and all cells, including those destined to become gonocytes and eventually spermatogonial stem cells, will have this same chromosomal constitution. As the spermatogonial stem cell precursors and stem cells themselves migrate to the gonadal ridge from the yolk sac, they expand mitotically in number and begin the slow process of differentiation ^12– 14^. Even after invading and populating the emergent seminiferous tubules, numerical increase continues until birth. With the onset of the mini-puberty (neonatal surge of gonadotropins) during the first few months of life, proliferation and differentiation (of some) to type A dark (Ad) spermatogonia commences and, when this temporally limited hypothalamic-pituitary stimulation stops, the spermatogonia become quiescent until puberty, although there may be a gradual diminution in the absolute numbers of spermatogonia in the first year of life ^15, 16^. The vast majority of these resting cells would be 47,XXY but, occasionally, it is thought the supernumerary X chromosome is lost during an earlier mitotic replication and the resultant spermatogonial stem cell or type Ad spermatogonia is, therefore, normally diploid (46,XY) ^17^. These are thought by many to be the cells that eventually, upon initiation of puberty, will be capable of completing the full process of spermatogenic differentiation (mitosis, meiosis, and spermiogenesis), their progeny being fully functional haploid spermatozoa. However, the more numerous, by orders of magnitude, 47,XXY spermatogonia suffer a meiotic block, arrest, and become apoptotic ^18^. Whether it is simply the trisomic state or the overexpression of X-linked, testis-expressed genes that lead to this demise is unclear, although the latter hypothesis has more evidence behind it ^19, 20^. The legacy of this self-destruction is wide swaths of the testicular parenchyma with seminiferous tubules that are empty ghosts or unrecognizable and fibrotic. By happenstance, every once in a while, a seminiferous tubule in which a 46,XY spermatogonium found itself at home survives, incubates, and cultivates the normal machinery responsible for complete spermatogenesis ^21^. So, if 47,XXY spermatogonia have no ability to birth whole spermatozoa, is there a reason to harvest and cryopreserve them before they become apoptotic early in puberty? The answer would appear to be “no”, as concluded by Oates ^22^. Indeed, with regard to fertility preservation in even younger males with KS, as Gies *et al.* cautiously posit, “given these controversies, banking testicular tissue from prepubertal KS boys should be performed only in a research framework” ^23^, even though parents of KS boys would be in favor of it ^24^.

## Is there a better chance of finding spermatozoa in adolescence than in adulthood?

Given the above discussion that spermatozoa arise from random 46,XY spermatogonia scattered about a sea of fibrotic tubules, the next obvious question in the search to answer whether it is advantageous to perform TESE in an adolescent KS male as opposed to waiting until adulthood would naturally be whether the chance of finding sperm upon TESE is greater in the adolescent than it is in the adult. If so, that would be a rationale for TESE in these younger males, but if not there would seem to be no benefit in doing so. That is, is there a great likelihood that these competent and capable 46,XY populated seminiferous tubules will also disappear during puberty or in the next several years, perhaps collateral damage of the near total annihilation of the neighboring 47,XXY spermatogonia and their home tubules and, thusly, they and any sperm they produce should be harvested and saved for the future as soon as possible?

Sperm seen within the ejaculate are probably only a small percentage of the total number produced in the testis when viewing the entirety of the testis parenchyma as a single manufacturing unit. When that output falls below a certain minimum number, but not to zero, no sperm can be found downstream in the seminal fluid, but some of the relatively few that have been created may still be identified when combing through and dissecting that testis tissue factory itself. Shortly following the introduction of intracytoplasmic sperm injection (ICSI), it was realized that testicular sperm was capable of fertilization, embryo development, and pregnancy. TESE quickly became the standard therapy offered to men with NOA of all types in an effort to find and use individual spermatozoa to achieve male genetic parenthood ^25– 31^. Retrieved spermatozoa could even be intentionally frozen prior to use in conjunction with ICSI, as first demonstrated by Oates *et al.*, and this is now routine clinical practice ^32^. In their recent systematic review and meta-analysis, Bernie *et al.* concluded that the sperm retrieval rate (SRR) was higher with micro-TESE (involving the use of an operating microscope) than with conventional TESE ^33^ and approximates 50–60%, depending upon the center and the surgeon. Tournaye *et al.* initially pioneered its application in adult KS men, and the first pregnancy was documented by Palermo *et al.* ^34, 35^. Numerous subsequent publications confirmed this initial proof of concept and added to the growing cumulative statistic of approximately 50–60% likelihood of sperm retrieval in this subpopulation of NOA men ^2^.

Damani *et al.* first reported in 2001 the use of TESE in an adolescent male in order to ‘preserve’ his fertility before he was started on spermatogenically suppressive testosterone replacement ^36^. The authors carefully presented their data and the decisions that led the patient and his parents to choose this approach in a cautionary tone, advocating only that TESE in the adolescent KS male needed more study and consideration before becoming viewed as routine, always beneficial, and standard. Building upon that idea, a handful of subsequent reports presented data on testis biopsy or TESE in very young/adolescent KS boys with varying results. In 2004, Wikstrom *et al.* reported that they were not able to find any spermatozoa in the histological preparations of single-site biopsies on 14 boys aged 10–14 years ^37^. They noted a minimum number of type A pale (Ap) and Ad spermatogonia in seven of the 14, all within the age range of 10–12.5 years, but no spermatogonia of any kind in the remaining seven who had an age range of 11.7–14 years. In 2012, Gies and colleagues, who also performed a single-site biopsy but of “large volume”, reported that they too could not document full spermatogenesis on histological analysis in seven KS boys, aged 10.2–15.6 years ^38^. Spermatogonia were seen in four of the seven in the upper age range of their group (13.3–15.9). Another seemingly disappointing report by Rivas *et al.* in 2013 stated that only one KS adolescent out of five operated upon had spermatozoa visualized, even though they carried out single-site bilateral TESE ^39^. Mehta *et al.*, also in 2013, using a microsurgical TESE approach reported positive news with sperm discovery in seven of 10 adolescents, ages ranging from 14–22 years: an SRR of 70% ^40^. Plotten *et al.* in 2015 described an SRR of 52% in “young” KS patients, aged 15–23 ^9^. Finally, Nahata *et al.* reported a 50% SRR in 10 adolescent KS boys aged 15–23 when performing microTESE ^41^. Sperm was identified in five KS boys (aged 16, 16, 17, 19, and 23) but was not discovered in the remaining five KS boys (aged 15, 16, 16, 18, and 20), indicating no real pattern or prediction based upon age at the time of TESE.

As above, SRR rates of TESE in KS adults approximate 50–60%. But are there data to compare within the same program, adolescent to adult? Plotten *et al.* performed exactly that study showing prospectively, as detailed previously, an SRR of 52% in their “young” group and 62% (no statistical difference) in their “adult” group, aged over 23 years ^9^. At Weill Cornell Medical College, Dabaja and Schlegel confined their reported results to 127 adult KS men and showed an SRR of 65%, which is similar to that of 70% (7/10) reported by Mehta *et al.* at the same institution ^40, 42^. As stated and suggested by Nahata *et al.* in their conclusion, “…there is no clear benefit to performing TESE/unilateral microTESE in adolescence…” ^41^. In total, then, although perhaps intuitively satisfying, sensible, and gratifying, SRRs in the small numbers of adolescent KS boys subjected to TESE appear to be the same as those SRRs in adult KS men subjected to TESE. In general, the sperm retrieved from the adult is used immediately as the source of sperm for ICSI and not cryopreserved. Will cryopreserved spermatozoa or testis tissue be equally as useable and successful after years of storage as would necessarily be the case if harvested from a young boy not ready to conceive for years? Future reports detailing the success of using this sperm (obtained many years previously) will be necessary to answer this question. This will include those adolescents who have had microTESE prior to the institution of spermatogenically suppressive exogenous testosterone therapy.

## Does sperm production decline in the years of adolescence and on into adulthood to negligible levels?

This is another way of asking the same question: should we be performing TESE in the adolescent to preserve fertility that will be irrevocably lost as the years pass and we do nothing? This notion also arises from very limited data suggesting that sperm production, as determined by results of TESE (namely SRR), declines after a certain age (35 or so) and as such it might be best to harvest tissue as soon as the diagnosis is made, whatever the age ^43^. Although these conclusions have been made based on a limited data set from Okada *et al.* ^43^ (25 patients greater than age 35 with a 23% SRR, far different from the Cornell group as detailed below), this is by no means certain. As a matter of fact, the data from the Cornell group ^44^ show that the SRR in KS men was 71% in the 22–30 year age group, 86% in the 31–35 year age group, and 50% in the 36–50 year age group. The group age intervals are not of the same time spans, and the SRR is higher when you are 33 than when you are 26 (how is that possible biologically?) and is still 50% when greater than age 36. As they state, “In addition, ROC analysis of these data yielded an AUC of 0.58, indicating that there is no best age to predict SRR based on our data”. So it appears that even in the publications discussing whether or not TESE rates are less in the ‘older’ adult than in the ‘younger’ adult (aged 35), there is still a rate of success that is close to, and not much better than, that claimed for adolescents.

Finally, when comparing overall adolescent rates to overall adult rates of TESE success, and although difficult to prove, there would be little reason to believe that sperm production would magically begin years after the onset of puberty, e.g. age 16 or 17. Therefore, since 50 out of every 100 KS boys have sperm recovered in their testis tissue and 50 out of every 100 KS adults have sperm recovered from their testis tissue, and if a decline in sperm production occurred during this time frame to zero values of, for argument’s sake, 10%, then 10% of maturing KS males (adolescent to adult ages) would have to begin to produce sperm to keep the two groups equivalent, as far as SRR rates go. Is it likely that 10 (or whatever absolute number is chosen) males have a precipitous and total decline in sperm production as they advance from adolescence into adulthood and, to keep the rates of what we actually find upon TESE balanced, 10 males begin to suddenly produce sperm as they advance from adolescence into adulthood? Or is it more likely that those who have seminiferous tubules populated by 46,XY spermatogonia capable of undergoing the full and complete sequence of spermatogenesis maintain those same tubules throughout the years of adolescence and on into adulthood? It may be the exact same individuals who have spermatozoa found in adolescence that are those found in adulthood. Where would the benefit be? Harm could certainly come to the adolescent and his parents when no sperm are retrieved—a potential heavy burden to carry in the difficult teenage years.

## Conclusion

The basic biology of spermatogenesis and how it is altered in the KS testis is important to appreciate when trying to formulate appropriate clinical pathways vis-à-vis fertility and biological paternity. Although it seems obvious to some that KS boys benefit or even require testis tissue surgery and freezing of any sperm found as soon as they are identified in the adolescent years, the data do not support the temporal necessity to do so. It may be just as reasonable, if not more so, to perform that same surgery at a later date, in adulthood when the patient and his partner can make an informed choice as to whether an operation on him and an *in vitro* cycle for her is what they would like to do in an effort to build a family. Since the incidence of KS syndrome is 1:600, practitioners of all types are going to have many KS males as their patients, will be involved in this debate and controversy, and will need to be aware of the issues involved. This list includes, but is not limited to, obstetricians (amniocentesis results), pediatricians (discovered at the time of a learning disability evaluation, or to understand the reason for small testes), endocrinologists (detected during exploration for hypogonadism and failure to initiate puberty in the most severely affected), urologists, and reproductive endocrinologists (elucidated at the time of an infertility work-up). As it usually is, the final answer will probably lie somewhere in the middle and be determined on an individual patient-specific basis.

## References

[1] Wikstrom AM Dunkel L: Klinefelter syndrome. *Best Pract Res Clin Endocrinol Metab.* 2011;25(2):239–50. 10.1016/j.beem.2010.09.006 21397196

[2] Majzoub A Arafa M Al Said S: Outcome of testicular sperm extraction in nonmosaic Klinefelter syndrome patients: what is the best approach? *Andrologia.* 2016;48(2):171–6. 10.1111/and.12428 25929757

[3] Herlihy AS Halliday JL Cock ML: The prevalence and diagnosis rates of Klinefelter syndrome: an Australian comparison. *Med J Aust.* 2011;194(1):24–8. 21449864 10.5694/j.1326-5377.2011.tb04141.x

[4] Manning MA Hoyme HE: Diagnosis and management of the adolescent boy with Klinefelter syndrome. *Adolesc Med.* 2002;13(2):367–74, viii. 11986043

[5] Mehta A Mielnik A Schlegel PN: Novel methylation specific real-time PCR test for the diagnosis of Klinefelter syndrome. *Asian J Androl.* 2014;16(5):684–8. 10.4103/1008-682X.125914 24923458 PMC4215686

[6] Radicioni AF Ferlin A Balercia G: Consensus statement on diagnosis and clinical management of Klinefelter syndrome. *J Endocrinol Invest.* 2010;33(11):839–50. 10.1007/BF03350351 21293172

[7] Tincani BJ Mascagni BR Pinto RD: Klinefelter syndrome: an unusual diagnosis in pediatric patients. *J Pediatr (Rio J).* 2012;88(4):323–7. 10.2223/JPED.2208 22915094

[8] Mehta A Paduch DA: Klinefelter syndrome: an argument for early aggressive hormonal and fertility management. *Fertil Steril.* 2012;98(2):274–83. 10.1016/j.fertnstert.2012.06.001 22732737

[9] Plotton I Giscard d'Estaing S Cuzin B: Preliminary results of a prospective study of testicular sperm extraction in young versus adult patients with nonmosaic 47,XXY Klinefelter syndrome. *J Clin Endocrinol Metab.* 2015;100(3):961–7. 10.1210/jc.2014-3083 25423570

[10] Salbenblatt JA Bender BG Puck MH: Pituitary-gonadal function in Klinefelter syndrome before and during puberty. *Pediatr Res.* 1985;19(1):82–6. 10.1203/00006450-198501000-00022 3918293

[11] Aksglaede L Skakkebaek NE Almstrup K: Clinical and biological parameters in 166 boys, adolescents and adults with nonmosaic Klinefelter syndrome: a Copenhagen experience. *Acta Paediatr.* 2011;100(6):793–806. 10.1111/j.1651-2227.2011.02246.x 21362037

[12] Davis SM Rogol AD Ross JL: Testis Development and Fertility Potential in Boys with Klinefelter Syndrome. *Endocrinol Metab Clin North Am.* 2015;44(4):843–65. 10.1016/j.ecl.2015.07.008 26568497 PMC4648691

[13] Hughes IA: Minireview: sex differentiation. *Endocrinology.* 2001;142(8):3281–7. 10.1210/endo.142.8.8406 11459768

[14] Magnon C Lucas D Frenette PS: Trafficking of stem cells. *Methods Mol Biol.* 2011;750:3–24. 10.1007/978-1-61779-145-1_1 21618080

[15] Mikamo K Aguercif M Hazeghi P: Chromatin-positive Klinefelter's syndrome. A quantitative analysis of spermatogonial deficiency at 3, 4, and 12 months of age. *Fertil Steril.* 1968;19(5):731–9. 10.1016/S0015-0282(16)36787-5 5676479

[16] Müller J Skakkebaek NE Ratcliffe SG: Quantified testicular histology in boys with sex chromosome abnormalities. *Int J Androl.* 1995;18(2):57–62. 10.1111/j.1365-2605.1995.tb00386.x 7665210

[17] Vialard F Bailly M Bouazzi H: The high frequency of sperm aneuploidy in Klinefelter patients and in nonobstructive azoospermia is due to meiotic errors in euploid spermatocytes. *J Androl.* 2012;33(6):1352–9. 10.2164/jandrol.111.016329 22492842

[18] Wikström AM Dunkel L: Testicular function in Klinefelter syndrome. *Horm Res.* 2008;69(6):317–26. 10.1159/000117387 18504390

[19] Johannisson R Gropp A Winking H: Down's syndrome in the male. Reproductive pathology and meiotic studies. *Hum Genet.* 1983;63(2):132–8. 10.1007/BF00291532 6220959

[20] Ross MT Grafham DV Coffey AJ: The DNA sequence of the human X chromosome. *Nature.* 2005;434(7031):325–37. 10.1038/nature03440 15772651 PMC2665286

[21] Sciurano RB Luna Hisano CV Rahn MI: Focal spermatogenesis originates in euploid germ cells in classical Klinefelter patients. *Hum Reprod.* 2009;24(9):2353–60. 10.1093/humrep/dep180 19443454

[22] Oates RD: The natural history of endocrine function and spermatogenesis in Klinefelter syndrome: what the data show. *Fertil Steril.* 2012;98(2):266–73. 10.1016/j.fertnstert.2012.06.024 22846647

[23] Gies I Oates R De Schepper J: Testicular biopsy and cryopreservation for fertility preservation of prepubertal boys with Klinefelter syndrome: a pro/con debate. *Fertil Steril.* 2016;105(2):249–55. 10.1016/j.fertnstert.2015.12.011 26748226

[24] Gies I Tournaye H De Schepper J: Attitudes of parents of Klinefelter boys and pediatricians towards neonatal screening and fertility preservation techniques in Klinefelter syndrome. *Eur J Pediatr.* 2016;175(3):399–404. 10.1007/s00431-015-2657-7 26494133

[25] Craft I Bennett V Nicholson N: Fertilising ability of testicular spermatozoa. *Lancet.* 1993;342(8875):864. 10.1016/0140-6736(93)92722-6 8104288

[26] Devroey P Liu J Nagy Z: Pregnancies after testicular sperm extraction and intracytoplasmic sperm injection in non-obstructive azoospermia. *Hum Reprod.* 1995;10(6):1457–60. 10.1093/HUMREP/10.6.1457 7593514

[27] Lewin A Weiss DB Friedler S: Delivery following intracytoplasmic injection of mature sperm cells recovered by testicular fine needle aspiration in a case of hypergonadotropic azoospermia due to maturation arrest. *Hum Reprod.* 1996;11(4):769–71. 10.1093/oxfordjournals.humrep.a019252 8671326

[28] Mulhall JP Burgess CM Cunningham D: Presence of mature sperm in testicular parenchyma of men with nonobstructive azoospermia: prevalence and predictive factors. *Urology.* 1997;49(1):91–5; discussion95–6. 10.1016/S0090-4295(96)00356-1 9000192

[29] Mulhall JP Reijo R Alagappan R: Azoospermic men with deletion of the DAZ gene cluster are capable of completing spermatogenesis: fertilization, normal embryonic development and pregnancy occur when retrieved testicular spermatozoa are used for intracytoplasmic sperm injection. *Hum Reprod.* 1997;12(3):503–8. 10.1093/humrep/12.3.503 9130751

[30] Schoysman R Vanderzwalmen P Nijs M: Pregnancy after fertilisation with human testicular spermatozoa. *Lancet.* 1993;342(8881):1237. 10.1016/0140-6736(93)92217-H 7901551

[31] Schoysman R Vanderzwalmen P Nijs M: Successful fertilization by testicular spermatozoa in an *in-vitro* fertilization programme. *Hum Reprod.* 1993;8(8):1339–40. 8408540 10.1093/oxfordjournals.humrep.a138254

[32] Oates RD Mulhall J Burgess C: Fertilization and pregnancy using intentionally cryopreserved testicular tissue as the sperm source for intracytoplasmic sperm injection in 10 men with non-obstructive azoospermia. *Hum Reprod.* 1997;12(4):734–9. 10.1093/humrep/12.4.734 9159434

[33] Bernie AM Mata DA Ramasamy R: Comparison of microdissection testicular sperm extraction, conventional testicular sperm extraction, and testicular sperm aspiration for nonobstructive azoospermia: a systematic review and meta-analysis. *Fertil Steril.* 2015;104(5):1099–103.e1-3. 10.1016/j.fertnstert.2015.07.1136 26263080

[34] Palermo GD Schlegel PN Sills ES: Births after intracytoplasmic injection of sperm obtained by testicular extraction from men with nonmosaic Klinefelter's syndrome. *N Engl J Med.* 1998;338(9):588–90. 10.1056/NEJM199802263380905 9475766

[35] Tournaye H Staessen C Liebaers I: Testicular sperm recovery in nine 47,XXY Klinefelter patients. *Hum Reprod.* 1996;11(8):1644–9. 10.1093/oxfordjournals.humrep.a019462 8921109

[36] Damani MN Mittal R Oates RD: Testicular tissue extraction in a young male with 47,XXY Klinefelter's syndrome: potential strategy for preservation of fertility. *Fertil Steril.* 2001;76(5):1054–6. 10.1016/S0015-0282(01)02837-0 11704135

[37] Wikström AM Raivio T Hadziselimovic F: Klinefelter syndrome in adolescence: onset of puberty is associated with accelerated germ cell depletion. *J Clin Endocrinol Metab.* 2004;89(5):2263–70. 10.1210/jc.2003-031725 15126551

[38] Gies I de Schepper J van Saen D: Failure of a combined clinical- and hormonal-based strategy to detect early spermatogenesis and retrieve spermatogonial stem cells in 47,XXY boys by single testicular biopsy. *Hum Reprod.* 2012;27(4):998–1004. 10.1093/humrep/des002 22313866

[39] Rives N Milazzo JP Perdrix A: The feasibility of fertility preservation in adolescents with Klinefelter syndrome. *Hum Reprod.* 2013;28(6):1468–79. 10.1093/humrep/det084 23539613

[40] Mehta A Bolyakov A Roosma J: Successful testicular sperm retrieval in adolescents with Klinefelter syndrome treated with at least 1 year of topical testosterone and aromatase inhibitor. *Fertil Steril.* 2013;100(4):970–4. 10.1016/j.fertnstert.2013.06.010 23830150

[41] Nahata L Yu RN Paltiel HJ: Sperm Retrieval in Adolescents and Young Adults with Klinefelter Syndrome: A Prospective, Pilot Study. *J Pediatr.* 2016;170:260–265.e2. 10.1016/j.jpeds.2015.12.028 26746120

[42] Dabaja AA Schlegel PN: Microdissection testicular sperm extraction: an update. *Asian J Androl.* 2013;15(1):35–9. 10.1038/aja.2012.141 23241638 PMC3739122

[43] Okada H Goda K Yamamoto Y: Age as a limiting factor for successful sperm retrieval in patients with nonmosaic Klinefelter's syndrome. *Fertil Steril.* 2005;84(6):1662–4. 10.1016/j.fertnstert.2005.05.053 16359961

[44] Ramasamy R Ricci JA Palermo GD: Successful fertility treatment for Klinefelter's syndrome. *J Urol.* 2009;182(3):1108–13. 10.1016/j.juro.2009.05.019 19616796

